# Effects of Ergot of Rye on the Parturient Female and Her Offspring

**Published:** 1845-12

**Authors:** Samuel Hardy


*On the Effects of Ergot of Rye on the Parturient Female and her Offspring.* By Samuel Hardy, M.D. Paper read before the Dublin Obstetrical Society.

Long as has been the practice of administering the secale cornutum as an obstetrical medicine, its effects, both salutary and the reverse, are far from being distinctly ascertained. The object of the communication, which we shall here condense, is that of rendering our knowledge upon the subject more precise. In carrying out this object, the author investigates the action of the medicine under the following different points of view:

1. *As to the time the action of the Ergot on the uterus commences.*—From certain tables, this time appears to be in some cases as early as seven minutes after its exhibition, while in others a much longer period is required; the average time appears to be about ten or fifteen minutes. The author considers that it has always commenced within twenty-five minutes at the furthest, when the child has been expelled alive. On the other hand, if a longer time than this has elapsed, instruments have been necessary, and the infant has been born dead. The beneficial action of the ergot is evidenced by the pains running one into another without any appreciable interval.

2. *Effect on the maternal pulse.*—This part of the inquiry is one of considerable interest, and has not received the attention from practitioners that it deserves. In 19 cases recorded by the author, there was a marked diminution in the frequency of the mother's pulse, after the administration of the ergot; and this effect generally commenced within fifteen minutes of its exhibition. In all cases in which the maternal pulse was affected, the fœtal heart underwent a corresponding change. Here a practical question naturally arises: is ergot a safe remedy in relaxation of the uterus, when the woman is reduced by previous hemorrhage?

The author does not give us any decided reply to this question, but contents himself with allusion to a single case in point, in which alarming prostration followed its exhibition.

3. *The effects of Ergot on the Fœtal Heart.*—This is said to be still more remarkable than the effect upon the maternal pulse, and therefore demands serious consideration. By reference to the tables, it will be found that in the majority of cases a diminution in the pulsation of the fœtal heart, followed the exhibition of ergot. The period at which this commences does not differ from that previously noticed, namely, fifteen minutes; the most usual effect noticed by the author is a diminution, in the first place, of the frequency of the pulsation, which is succeeded shortly by irregularity in the beats, or complete intermission. The author here states a practical fact, deduced from his own observations, to the effect that the child is generally lost, however speedily the delivery be completed, if the pulsation of the fœtal heart are reduced to 110, and at the same time become *intermittent.* The intermissions are a point of great importance in this statement, for the reduction of the pulse below 110 without this concomitant is not necessarily a fatal symptom.

Many different opinions have been broached as to the modus operandi of the ergot in destroying the life of the fœtus, some attributing it to the powerful compression exercised by the uterine walls, others to a specific poisonous effect of the medicine. The author thinks that each opinion may, to a certain extent, be correct, but leans evidently to that which attributes it to the poisonous qualities of the ergot.

The depressing effects of ergot upon the fœtal heart are so great, that a considerable time elapses after birth before the child can be restored. The author has observed that children equally weak are restored to animation with much less difficulty when ergot has not been given.

The author, in alluding to the proper time at which the ergot should be given for the purpose of restraining or preventing 'post partum' flooding, states, that there are three periods at which the medicine may be administered;—first, when the head is about to pass; secondly, after it has been expelled; and thirdly as soon as the index-finger can reach the insertion of the funis into the placenta.

4. *The state of the uterus and lochial discharge.*—After the use of ergot, the uterus has frequently been found much larger than in ordinary labors, as has been remarked also by Dr. Johnson. The lochial discharge was sometimes pale and scanty. The children which are born alive usually do well.

The mode of administration of the ergot varies with different practitioners. The plan adopted by the author is to infuse half a drachm of the powder in three ounces of boiling water, and after straing to add ten or fifteen grains of fresh powder with a little sugar. This dose is repeated in twenty minutes, and if the uterus does not contract well is given a third time.—*Ibid.*

